# A corona virus tracker for clinicians and students: Assessing education during an evolving phenomenon

**DOI:** 10.15694/mep.2020.000111.1

**Published:** 2020-05-26

**Authors:** Kacper Niburski, Oskar Niburski

**Affiliations:** 1 McGill University; 2 York University

**Keywords:** coronavirus, covid-19, medical education, web application, epidemiology

## Abstract

This article was migrated. The article was marked as recommended.

## Letter

In a novel pandemic, knowledge is paramount to ensure evidence-based, appropriate treatments. Unfortunately, the sources of information in an evolving situation are varied, often influenced by pseudo-science, and can be sensationalized ( Geldsetzer, 2020). Furthermore, there are few sources that teach the findings in ways accessible to both the public and clinicians.

### Methodology

We created a tracker ( whohascoronavirus.com) on March 1 ^st^ to teach treatment and the epidemiology of COVID-19 to healthcare practitioners and general public. Daily briefings from the WHO were collected, displaying death rates, recovery rates, active cases, and total cases of respective countries, individual regions, and states. Clinical information regarding treatment modalities, radiographic images, and was summarized from the Journal of the American Medical Association (JAMA). Ethics exemption was received from Ilde Lepore, McGill Medicine’s Institutional Review Board ethics officer, due to the public availability of the information and the lack of reasonable expectation of privacy on a public website with public information.

### Results

Over the months, thousands visited the site, with an average of 5.2 minutes on site. 250 users were sampled randomly within a week (April 1 - April 8). 58% (n=145) reported to be healthcare practitioners, with 68% (n=98) being medical students, 22% (n=33) nurses, 10% (n=14) clinicians, and the rest being the general public. On a scale from 1-5, 5 being very improved, healthcare practitioners reported improvement in general knowledge (2.3 ± 0.4 to 4.4 ± 0.6, p<0.01), improvement in understanding of the epidemiological situation of the corona virus (1.8 ± 1.1 to 4.5 ± 0.3, p<0.01), improvement in clinical treatment (3.4 ± 0.4 to 4.0 ± 0.3, p<0.05), with the most observed increases in medical students and nursing. General public saw similar increases in general knowledge (1.1 ± 0.9 to 3.7 ± 0.5, p<0.01). Both groups (n=90, 62% in healthcare, n-82, 78% general population) stated that having knowledge made them feel less anxious about the pandemic. 90% of total users (n=225) stated they’d visit the site again the following day, to continue monitoring information and growth of COVID-19.

### Discussion

These results are the first to detail the findings of a web educational initiative for COVID-19. They show that when facts are appropriately sourced and manageably displayed, there is greater understanding in both clinician and general public. These gains are seen most in medical students, as well in lay comprehension of the novel pandemic.

During times of uncertainty and need for knowledge, such a resource could help provide clues on how to best educate on an evolving situation. Unproven treatments tend to get airtime, due to both controversy and influence ( Liu *et al.,* 2020). As a result, technologies have to be limber, worked with daily, and enable the highest level of accurate information. The future work of education must address the flexibility necessary for a changing situation by itself being limber. Such data would provide a fuller picture of the pandemic and allow clinicians to learn as treatment modalities change and resources become stretched.

## Notes On Contributors

Kacper Niburski is a third year medical student at McGill University. He is interested in how technology can be used to increase medical education. See his work at: https://orcid.org/0000-0003-1519-1842. Oskar Niburski is a software engineer working at the forefront of tech. He is interested in discovering new ways of analyzing data, to provide evidence-based decisions.
