# Supplementary material for: A corona virus tracker for clinicians and students: Assessing education during an evolving phenomenon
Source: MedEdPublish (2016). 2020 May 26;9:111. [Version 1] doi: 10.15694/mep.2020.000111.1 (PMC10702659; doi:10.15694/mep.2020.000111.1)
Supplement: Peer review history

# Reviewer report: Reviewer response for version 1

**DOI:** 10.21956/mep.20082.r30913

**Published:** 2020-06-11

**Authors:** Richard Hays

**Affiliations:** 1 James Cook University

This review has been migrated. The reviewer awarded 3 stars out of 5 I found this an interesting report, although I am not sure that the idea was new and I am not sure what other apps were available at the time. Was this the first such source available? Where I live, a similar app was released by the Government at about the same time so that all citizens could gain access more easily to the latest local, national and international information. It is useful, although my professional networks send me daily updates on relevant clinical information so I have not often needed to use the app. This app seems to be a combination of the two approaches, with more clinically oriented information. It would be interesting to see longer term usage data. It may well prove to be useful beyond the medical school system in which was developed.

Reviewer Expertise:

NA

No decision status is available

# Reviewer report: Reviewer response for version 1

**DOI:** 10.21956/mep.20082.r30914

**Published:** 2020-06-02

**Authors:** P Ravi Shankar

**Affiliations:** 1 American International Medical University

This review has been migrated. The reviewer awarded 4 stars out of 5 This is an interesting letter describing the ‘visitors’ perception of a coronavirus tracker (www.whohascoronavirus.com). The authors have put together a dynamic website to track the rapidly evolving pandemic. The graphs are interesting and up-to-date. The authors have a button to toggle between information for patient and information for clinicians which I think is a cool feature. The site provides basic information about the pandemic. The numbers however in the tables regarding total cases and total deaths may not be current. Also, in the ensuing three months since the site was first developed the distribution of cases globally has changed with the United States, Russia, Turkey and developing countries like Brazil, India and Peru accounting for an increasing number of cases. I am sure the authors may find keeping the website updated a challenge and will be interested in knowing how they do so. This is a good initiative and readers will find it to be of interest.

Reviewer Expertise:

NA

No decision status is available

# Reviewer report: Reviewer response for version 1

**DOI:** 10.21956/mep.20082.r30916

**Published:** 2020-05-27

**Authors:** Felix Silwimba

**Affiliations:** 1 University of Lusaka

This review has been migrated. The reviewer awarded 5 stars out of 5 it is important to keep information flowing and tracking it. this was a goof initiative

Reviewer Expertise:

NA

No decision status is available

# Reviewer report: Reviewer response for version 1

**DOI:** 10.21956/mep.20082.r30915

**Published:** 2020-05-26

**Authors:** Virginia Randall

**Affiliations:** 1 Uniformed Services University of the Health Sciences

This review has been migrated. The reviewer awarded 5 stars out of 5 I was glad to see this website created and used to provide healthcare practitioners with accurate information. I went to the website and it displays the information in clear charts and graphs and does not have an overwhelming amount displayed. But, I wish it had an update on treatment and prevention written for the practitioners (here is where it seems the most pseudoscience is published). I would also liked to have seen the results presented as a table rather than in a written paragraph. I'm left wondering what the response rate was of their sample, how many have visited the site, but most of all, how many man hours is it taking to keep the statistics up to date for so many countries?? This is a great way to push out accurate information quickly. And it certainly is true, accurate information is important to reduce anxiety and put fear into context. It is a good model for dissemination of accurate information quickly, but relies on availability of the internet and the capability and man hours to keep it up to date.Publicizing would be important (I teach in a medical school and this is the first of I seen this website.)

Reviewer Expertise:

NA

No decision status is available
